# Sclerostin Modulation Holds Promise for Dental Indications

**DOI:** 10.3390/healthcare6040134

**Published:** 2018-11-23

**Authors:** Mohamed G. Hassan, Abbas R. Zaher, Juan Martin Palomo, Leena Palomo

**Affiliations:** 1 Division of Craniofacial Anomalies, Department of Orofacial Sciences, University of California San Francisco, San Francisco, CA 94143, USA; mgamal@dent.svu.edu.eg; 2 Department of Orthodontics, Faculty of Dentistry, Alexandria University, Alexandria 21526, Egypt; azaher@drabbaszaher.com; 3 Department of Orthodontics, Faculty of Oral and Dental Medicine, South Valley University, Qena 83523, Egypt; 4 Department of Orthodontics, School of Dental Medicine, Case Western Reserve University, Cleveland, OH 44106-4905, USA; palomo@case.edu; 5 Department of Periodontics, School of Dental Medicine, Case Western Reserve University, Cleveland, OH 44106-4905, USA

**Keywords:** sclerostin, anti-sclerostin, bone remodeling, alveolar bone, orthodontic, tooth movement, periodontitis, bone loss

## Abstract

Sclerostin modulation is a novel therapeutic bone regulation strategy. The anti-sclerostin drugs, proposed in medicine for skeletal bone loss may be developed for jaw bone indications in dentistry. Alveolar bone responsible for housing dentition share common bone remodeling mechanisms with skeletal bone. Manipulating alveolar bone turnover can be used as a strategy to treat diseases such as periodontitis, where large bone defects from disease are a surgical treatment challenge and to control tooth position in orthodontic treatment, where moving teeth through bone in the treatment goal. Developing such therapeutics for dentistry is a future line for research and therapy. Furthermore, it underscores the interprofessional relationship that is the future of healthcare.

## 1. Introduction

The idea of pharmacologic alveolar bone modulation as a strategy to treat oral conditions is not new, nor is it a new concept to apply existing drugs for new indications Alveolar bone and skeletal bone remodeling share similar mechanisms. Both involve osteoblast and osteoclast balance regulated through signaling systems involving common hormones, cytokines and pathways. There are also important differences between alveolar bone and skeletal bone remodeling. Although these have not been well elucidated through well controlled studies, it is acceptable to say that Bisphosphonate drugs have been used this way [1]. Historically, bisphosphonates were in wide use for prevention and treatment of osteoporosis, Paget’s disease, metastatic bone conditions and conditions where cytokine over activity leads to upregulation in osteoclasts [2,3,4]. Bisphosphonates reduce net bone loss related to high bone turnover rates by interfering with osteoclasts [2,5]. Strategically targeting the osteoclastic resorption process is the pharmacologic niche for Alendronate and Risedronate to slow osteoporotic bone resorption in otherwise healthy postmenopausal women and people on long term corticosteroids. Other bisphosphonates, given in much higher doses through IV, such as Pamidronate were mainly used in cancer indications to limit bone pain secondary to metastasis [6,7].

However, even as bisphosphonates were heralded for their anti-skeletal fracture outcomes in the case reports emerged linked them to a negative side effect called bisphosphonate related osteonecrosis of the jaws (BRONJ) [8,9]. Although it was rare, BRONJ is very severe, disfiguring and has high rates of morbidity and mortality [10]. While BRONJ, its infancy as a condition, was being studied and defined it was noted that daily compliance related to oral dosing for prevention and treatment of postmenopausal osteoporosis became a limitation, Zolendronate evolved from the cancer related indication for this purpose in a twice yearly bolus [11,12]. Related to BRONJ, well controlled multi-worldwide randomized placebo trials found Zolendronate to safe even when given through IV administration twice yearly [13]. The bisphosphonate strategy in dental applications in the alveolar bone met its limit due to the severe nature of BRONJ [14], however the concept of host modulation of alveolar bone in dental applications continued to grow [15,16]. 

Although the bisphosphonates for dental applications were limited due to the severity of BRONJ, other drugs with similar capacity to regulate bone turnover, such as those from the sclerostin family, may be useful for dental applications. Dental applications in orthodontics and periodontics can conceptually benefit from modulating bone turnover. The aim of this article is to report on potential dental indications for sclerostins. 

Sclerostin modulating drugs are known for their most common indication, osteoporosis. They are also used to treat lesser known diseases such as sclerosteosis, loss of function of the gene encoding for sclerostin and Van Buchem’s disease, deletion of a downstream promoter of the sclerostin gene. Sclerostins are being heavily researched for many reasons. Since bone turnover modulation shares similar pathways and signaling systems not just in alveolar and skeletal bone, these drugs hold potential indications for several diseases/conditions including rheumatoid arthritis, bone health complications in diabetes and osteogenesis imperfecta. Intravenous administration is the most common route of administration but phase III and IV studies on other routes continue. 

Sclerostin is a negative osteoblast regulator. Sclerostins open the door modulating bone turnover through signaling agents which regulate bone homeostasis. The regulation of osteocyte-specific genes plays a major role in the process of bone remodeling common to both skeletal and alveolar bone [17,18]. Using their processes, osteocytes can communicate with cells on the bone surface and in the bone marrow [19,20]. Sclerostin, one of the proteins by which osteocytes regulate the function and number of the cells responsible for remodeling, is the product of the SOST gene [21,22]. Investigations of sclerostin deprived systems show enhanced bone formation. Altered sclerostin expression and restored bone formation after treatment with anti-sclerostin antibody in postmenopausal women and animal models suggest that sclerostin inhibition may be a viable approach for developing novel anabolic agents for diseases characterized by bone loss [23,24,25,26,27]. Sclerostin antibody (Scl-Ab) is receiving increasing attention as a bone-forming agent, as supported by studies in animals in which significant increases in bone volume and whole bone mechanical strength have been noted and in clinical trials showing increases in systemic bone formation markers and bone mineral density (BMD) [27,28,29,30]. The use of Scl-Ab leads to an increase in both osteoblast activity and osteoblast number, resulting in enhanced bone formation [31,32]. Studies with Scl-Ab treatment have demonstrated that osteoid synthesis and deposition appear to be upregulated globally as assessed by serum markers including P1NP (serum type 1 pro-collagen C-terminal/N-terminal) and osteocalcin [33]. Sclerostin secreted exclusively by osteocytes, is a glycoprotein that binds low-density lipoprotein receptor-related protein 5 and blocks the Wnt signaling pathway [34]. It suppresses osteoblastogenesis and reduces the viability of osteoblasts and osteocytes, leading to unbalanced bone turnover in favor of bone resorption not only by antagonizing Wnt but also by blocking bone morphogenetic protein signaling, both are essential for the maintenance of osteoblastogenesis [35,36,37]. Accordingly, it was shown that mechanical stimulation in vivo reduced the osteocytic sclerostin expression [38]. Also, it is possible that osteocyte death is a signal for bone formation because the level of sclerostin would decrease. Sclerostin levels are increased in mechanical unloading, aging and menopause, whereas they are decreased in hyperparathyroidism [39,40].

Adverse reactions at drug injection site have been noted. But the greater concern is to explain and understand the safety concerns that come with manipulation of the WNT pathway, because of its involvement in multiple cell functions. There is a potential for excess bone formation which includes potential to develop entrapment palsies, increased intracranial pressure and osteosarcoma. Furthermore, growing evidence suggests that WNT signaling inhibitors may contribute to chronic kidney disease-associated bone mineral disorder [41,42].

## 2. Orthodontic Tooth Movement

Orthodontic tooth movement (OTM) is considered as organized sterile inflammatory process, associated with the bone remodeling cascade [43]. Bone remodeling is necessary for OTM to occur. This unique cascade includes the osteoclast activation in areas where orthodontic pressure is applied. Here, localized bone resorption must necessarily occur in order for tooth movement. In this case, resorption is not a bad thing. On the tension areas of the tooth, counter to the force application, the opposite process occurs, as osteoblast action outweighs osteoclast action. There is bone formation [44,45,46,47].

**Figure 1:**
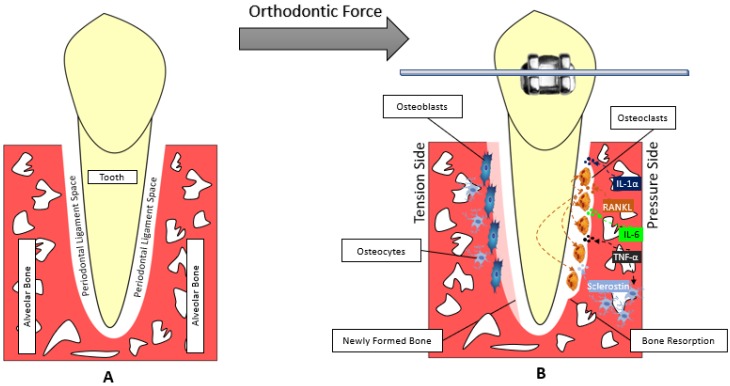
Model of the orthodontic tooth movement. (**A**) Before applying orthodontic force. (**B**) Applying orthodontic force to the tooth compresses the PDL. The compressed side of periodontal ligament is called the pressure side and the side where PDL is pulled is called the tension side. At the pressure side, Bone resorption is carried out mainly by osteoclasts and the help of other chemical mediators like sclerostin.

Bone change associated with both pressure and tension areas in OTM involve a complicated communication between three main tissues: alveolar bone, periodontal ligament, cementum (Figure 1) [48]. The signaling pathways, chemical mediators and signaling cytokines useful in alveolar bone remodeling needed for OTM are the same as those in skeletal bone remodeling. The expression of PDL cytokines and chemical mediators has been reported to be significantly altered during OTM. The levels of IL-1α, IL-1β, IL-6, IL-10, IL-17, IL-33 and TNFα are significantly increased shortly after force application, except IL-6 that remains high after 12 days then it starts to decrease to its normal levels after 3 weeks of force application [49,50,51,52,53,54,55,56,57,58].

The alteration of the PDL microenvironment (cytokines and chemical mediators) regulates the formation and function of osteoclasts [59]. In alveolar bone remodeling, just as in skeletal bone, there are two factors are controlling the osteoclastogenesis; the first is the RANKL and the second is macrophage colony-stimulating factor (M-CSF). RANKL is a downstream regulator of osteoclast formation and activation, through which cytokines produce their osteoresorptive effect. RANKL levels shortly increase after force application. Later, the increased RANKL expression is accompanied by upregulation of RANK expression, this RANK upregulation remains for 3 days following OTM. This binding leads to rapid differentiation of hematopoietic osteoclast precursors to mature osteoclasts [60,61].

**Figure 2:**
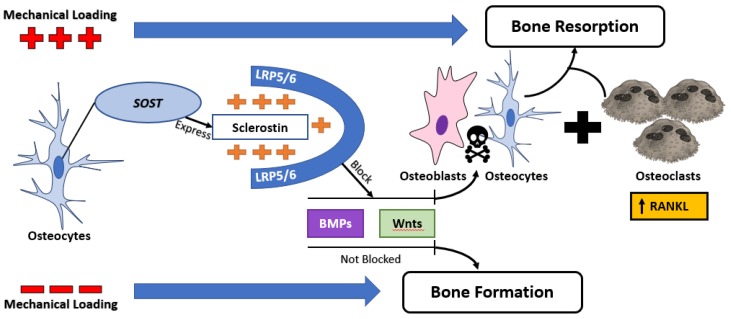
Relationship between mechanical loading and sclerostin expression. Mechanical loading upregulates sclerostin expression, leading to an inhibition of canonical Wnt signaling and exaggerate bone loss by upregulating osteoclastogenesis through the RANKL pathway.

Recently, the role of osteocytes during OTM has been well documented. The osteocytes in bone are thought to orchestrate “mechanotransduction” by reacting to various forms of mechanical stimulations through biologic cascades; this could be done by altering their sclerostin releases. In this way OTM manipulates bone loss in a useful way, such that tooth movement can occur. Bone loss in the OTM context is a good outcome such that tooth movement can occur into the site of bone resorption. (Figure 2). The response of the osteocytes to strain in vitro is controlled by the production of various chemical mediators like nitric oxide, prostaglandins, TNF-α and sclerostin. This mechanism is responsible for the activation of the PDL cells and the differentiation of precursors into osteoblasts or osteoclasts. Osteocyte response to mechanical loading during OTM using osteocyte knocked-out mice has been demonstrated, the changes in the level and the distribution of sclerostin in the PDL during OTM and linked it to the associated bone remodeling [62,63,64]. Given all the understanding of sclerostin and sclerostin-antibody pathways, we need to imply this knowledge in solving some of frequent problems occur during and/or after orthodontic treatment [65]. For example, is it possible to manage root wounds associated with orthodontic therapy using sclerostin-antibody treatment? Can we use the post-operative administration of anti-sclerostin to minimize teeth relapse and enhance retention? Is it possible and safe to use sclerostin or anti-sclerostin to accelerate or decelerate OTM? Well controlled studies are needed to answer these questions in an effort to introduce, efficacious, risk-free orthodontic therapy.

## 3. Periodontal Therapy

Destruction in periodontal disease occurs in the supporting structures of the teeth, namely: gingiva, periodontal ligament, cementum and alveolar bone, through a complex pathogenic process starting with the interaction between bacterial plaque biofilm and the host immune response. Gingivitis, the initial phase, is a reversible inflammatory response to biofilms. Gingivitis is limited to soft tissues adjacent to teeth. If unchecked gingivitis inflammation progresses first to the periodontal ligament then to the supporting alveolar bone. With progressive bone destruction, tooth mobility ensues and finally the tooth is lost. Reconstructing these bone defects is the periodontists treatment challenge, often dealt with using surgical procedures such as grafting and tissue regenerative strategies. 

Periodontitis and osteoporosis are both highly prevalent diseases associated with bone destruction [66]. Additionally, the two diseases have been considered as having overlapping pathogenesis and osteoporosis is a risk factor for the progression of periodontal destruction and potentially tooth loss [67]. The difference between osteoporosis and periodontitis is that periodontitis etiology involves host inflammation triggered by the build-up of bacterial plaque biofilm. This inflammation leads to subsequent loss of periodontal ligament, cementum and alveolar bone, the attachment apparatus of the tooth. Tissue destruction takes place in the perivascular extracellular gingival matrix. Here inflammation breaks down the collagen (Type I and III) and gingival proteoglycans. As inflammation spreads apically down the dental root surface, alveolar bone turnover shifts in favor of breakdown. In health, supporting alveolar bone is 1.5–2 mm apical to the cemento-enamel junction. In disease the distance is increased, the tooth is less supported and as a result more susceptible to abscess, trauma and ultimately tooth loss. 

In the alveolar bone component, osteoclasts, triggered by mast cells, neutrophils, macrophages, lymphocytes and plasma cells, through inflammatory cytokine mediators such as IL-1, -6, -8, -10 and TNF-alpha are responsible for alveolar bone resorption. These same cytokines are implicated in mediating bone loss in both postmenopausal osteoporosis and periodontitis [68]. One important difference between alveolar bone loss through this mechanism versus skeletal bone loss through this mechanism is that in alveolar bone loss due to periodontitis, local inflammatory response to bacteria on dental surfaces, upregulates the cytokine mediators. 

Osteocytic sclerostin expression has been linked to alveolar bone resorption during suppressed bone formation in rats with ligature-induced periodontitis. Kim et al. divided the rats into control and periodontitis groups. At 1, 3, 10 and 20 days after ligature, histologic an analysis of alveolar bone was performed and the numbers sclerostin-positive osteocytes were estimated, respectively. They noticed that sclerostin expression is increased when osteoid formation is suppressed. This may reinforce the hypothesis of the role of osteocytes as source of sclerostin during periodontitis-induced alveolar bone loss [69]. Clinical studies verified the results obtained from the animal studies regarding the involvement of sclerostin in alveolar bone loss associated with periodontitis. Sclerostin and sclerostin-RANKL ratio was found to be significantly lower in gingival crevicular fluid samples of healthy individuals than with patients with periodontitis [70].

The involvement of sclerostin in the inflammatory process associated with periodontitis and resulting in alveolar bone loss made many researchers to investigate the effect of the sclerostin antibody (Scl-Ab) on bone healing and the management of alveolar bone loss associating periodontitis. Animal trials showed that removal of SOST or blocking the expression of sclerostin reduces significantly bone loss associated with periodontitis in mouse models [71].

Due in part to these similarities between conditions, the concept of using same or similar pharmacotherapeutic intervention for both conditions is not new. The bisphosphonate family of medications, have been proposed as modulators of host bone loss resulting in pathologic amounts of bone loss. Farther upstream, at the macrophage level in the osteoclast activation cascade, parathyroid hormone is being explored to alter macrophage plasticity in tissues other than bone should be explored to treat bone loss in a locally delivered surgical periodontal treatment [72]. Similarly, NSAIDS and doxyclycline in sub anti-microbial doses, has been shown to modulate the host inflammatory response to down regulate collagen destruction [73,74].

The sclerostins fit this conceptual treatment model. Agents which inhibit sclerostins, such as anti-sclerostin antibodies show therapeutic outcomes such as increased bone formation, bone mass and density. Sclerostin inhibition using such neutralizing monoclonal antibodies have been proposed for postmenopausal osteoporosis and considered in preclinical models of osteogenesis imperfecta, rheumatoid arthritis and bone repair [75]. In the very same way, current findings show promise of sclerostins modulating oral bone related scenarios. Early animal studies show rats with a genetic sclerostin deficiency show increased jawbone growth. Additionally, in an experimental animal model sclerostin antibody administration reverses alveolar bone loss [76]. Furthermore, sclerostin immunolocalization has been demonstrated in periodontal tissues other than bone. Both cementocytes and periodontal ligament cultures show increasing sclerostin protein when adjacent tissues were exposed to cells genetically capable of producing sclerostins. As such both periodontal ligament cells and cementocytes show the potential participate in bone turnover when modulating osteocytes through sclerostin binding [77]. The critical question remains in what delivery system and in what dosages. More focused investigation into these approaches is to be expected.

## 4. Conclusions

Modulation of the alveolar bone has been discussed as the way of the future in dentistry. In periodontitis, alveolar bone loss can be countered using a pharmacotherapeutic if sclerostin therapy proves efficacious. This could be adjunctive to the conventional mechanical therapy involving the removal of pathologic biofilms accumulated on dental surfaces. In orthodontics, it can be used to influence tooth movement, either adjunctive to current treatment regimens using braces or aligners or plastic or metal retainers. 

Regulating alveolar bone turnover is important in several dental disciplines. In periodontics, reducing this turnover is a strategic means to prevent and treat disease. In orthodontics, modulating turnover can increase tooth movement or decrease relapse. Pharmacologic intervention in modulating host response in dentistry fits within the conceptual framework in which other drugs such as bisphosphonates have been attempted but side effects limited the utilization. Skeletal bone and alveolar bone share some common bone turnover mechanisms. Sclerostin is a central intracellular signaling protein with the ultimate effect of inhibiting osteoblastic bone formation. Regulating sclerostin is a novel way to modulate alveolar bone. This is one more example of interprofessional collaboration. Dentistry can borrow from medicine to the benefit of its patients.
